# Harnessing Both Phase Change and Isomerization: High-Energy-Density Azobenzene-Composites for Efficient Solar Energy Storage

**DOI:** 10.3390/molecules31010115

**Published:** 2025-12-29

**Authors:** Yan Jiang, Jiawei Chen, Yupeng Guo, Rui Liu, Hai Wang, Jin Huang, Wen Luo

**Affiliations:** 1 School of Mechanical and Automotive Engineering, Zhaoqing University, Zhaoqing 526061, China; jiangyan128@zqu.edu.cn (Y.J.); chenjw0203@163.com (J.C.); guoyupengwork@163.com (Y.G.); lruiooo2022@163.com (R.L.); 2 School of Electronic and Electrical Engineering, Zhaoqing University, Zhaoqing 526061, China; 3 School of Electric Engineering, Guangdong Polytechnic of Water Resources and Electric Engineering, Guangzhou 510635, China; huangjin@gdsdxy.cn; 4 School of Materials and Energy, Guangdong University of Technology, Guangzhou 510006, China

**Keywords:** organic phase change composite, azobenzene, solar energy storage, photo-isomerization, high-energy

## Abstract

Organic phase change materials (OPCMs) show immense application potential in solar energy storages owing to high energy storage capacity and latent heat efficiency. However, it is difficult to achieve prolonged energy storage due to the sensitivity of phase change to environmental temperature, and adding other substances will lead to a decrease in total energy density. Herein, azobenzene organic phase change composite (C_14_Azo-MA) was designed and prepared by doping myristic acid (MA) with an azobenzene derivative (C_14_Azo) featuring a carbon chain identical to that of the MA matrix. C_14_Azo-MA was systematically characterized by UV–Visible absorption spectroscopy and differential scanning calorimetry. The results showed that the C_14_Azo-MA retains the same isomerization properties as the C_14_Azo dopant. C_14_Azo-MA, due to its molecular photoisomerization and enhanced intermolecular interactions, establishes a new energy barrier and forms supercooling within C_14_Azo-MA, thereby allowing the storage of thermal energy below the crystallization temperature of MA. Notably, the C_14_Azo-MA exhibits a high energy density of 225.08 J g^−1^, surpassing that of pure MA by 14.42%. This work holds significant potential for solar energy storage applications.

## 1. Introduction

Due to the abundance and wide distribution of solar energy resources, as well as its clean and pollution-free characteristics, the application prospects of solar energy in the fields of renewable energy and clean energy are extremely broad [1,2,3]. In recent years, with the increasing global demand for sustainable energy, the development and utilization of solar energy have been increasingly focused upon by researchers. However, challenges are presented by the intermittent and unstable nature of solar energy, especially in matching energy supply with fluctuating demand across different times and regions [4,5,6]. Therefore, the development of efficient solar energy storage technologies is regarded as essential for achieving rational energy allocation and enhancing the efficiency of its utilization [7,8].

In order to effectively obtain and utilize solar energy, a large number of material systems have been proposed in recent years. These systems range from inorganic binary oxide ceramics (such as TiO_2_, ZnO, Al_2_O_3_) for photovoltaic and related applications [9] to organic photoswitchable molecules (known as molecular solar thermal (MOST)) [10,11] and organic phase change materials (OPCMs) for solar thermal energy storage [12]. Among them, due to their advantages in high energy storage capacity and latent heat efficiency, OPCMs have attracted increasing attention from experts and scholars [13,14,15,16]. OPCMs, classified as alkanes, fatty alcohols and acids, can absorb or release a large amount of thermal energy during the phase transition process, and thus are suitable for extensive applications across a wide range of temperatures [17,18,19], such as cold thermal energy storage [20,21,22], electronics/battery thermal management [23,24,25] and waste heat recovery [26,27,28]. However, traditional OPCMs often face rapid heat loss, reduced energy density lowered by the addition of other substances, and uncontrolled energy release (as the crystallization process is primarily influenced by ambient temperature). These issues severely limit their effectiveness in practical applications like long-duration solar thermal storage, efficient long-range heat transport and responsive on-demand heat delivery, particularly in regions experiencing limited sunlight or significant diurnal temperature swings [1,29,30]. Therefore, eliminating or avoiding the aforementioned issues of OPCMs has become a key focus of current research.

In recent years, the introduction of a new approach based on azobenzene (AZO) photo-switches into OPCMs has garnered increasing research interest, offering controllable and high energy density capabilities [7,31]. AZO, which can be also applied in the fields of liquid crystals [32,33], typically exhibits two distinct conformations: a stable *trans*-AZO at room temperature and a high-energy metastable *cis*-AZO. At ambient temperatures, AZO absorbs light energy, such as ultraviolet (UV) irradiation, and undergoes *trans*→*cis* photoisomerization, effectively storing energy as the isomerization enthalpy (Δ*H*_iso_, the energy difference between the two conformers). When it is integrated into OPCMs, the high-energy *cis*-AZO produced through *trans*→*cis* isomerization can induce photothermal supercooling, usually called photoinduced supercooling, which is characterized by a significant photoinduced crystallization temperature difference (Δ*T*_c_). This phenomenon allows OPCMs to remain stable in their liquid phase below the temperature of their intrinsic crystallization point, thereby achieving prolonged energy storage. Subsequently, exposure to visible light or elevated temperature can trigger the *cis*→*trans* photoisomerization reaction of high-energy metastable *cis*-AZO, releasing the stored energy as heat. Therefore, the combined release of the latent heat of OPCMs and the isomerization enthalpy of AZO is precisely controlled by visible light or thermal input, resulting in an increase in the overall energy density.

Han et al. [34,35] introduced AZO molecules into OPCMs for the first time. They demonstrated that the reversible isomerization of AZO groups under light irradiation changed their interactions with OPCMs, thus affecting the crystallization behavior of OPCMs. Their work achieved liquid-phase retention below the crystallization point of the original OPCMs and heat storage at 36 °C for about 10 h. On this basis, other researchers have explored more diverse molecular structure designs to optimize light control performance. Feng et al. [36] and our group [7] developed a series of photo-responsive composite phase change materials. These materials are based on azobenzene (AZO) as the core, grafted with alkyl ether or alkyl ester groups and then compounded with corresponding alkanes or alkanols. The studies have shown that these composites not only achieve an adjustable Δ*T*_c_ ranging from 3.33 to 8.80 °C, but also enable rapid simultaneous release of latent heat and photothermal energy (207 or 239 J g^−1^), while offering heat storage capabilities for up to 10 h. Chen et al. [31] systematically studied the light-controlled phase change ability of various grafted azobenzene derivatives on organic phase change materials. Their findings indicated that azobenzenes with altered polarity had a more significant impact on the crystallization behavior of fatty acid-based systems. Our group [8] also investigated the photo-controlled energy storage and thermal release of azobenzene-modified dodecanoic acid phase change composites. The results showed that composites with matching chain lengths exhibited optimal performance, with a tunable Δ*T*_c_ of 4–12 °C, and a synchronous heat release of 217.72 J g^−1^ under near-ambient temperature conditions. However, the relatively low energy density remains a limitation in practical applications. Furthermore, there is a scarcity of research specifically focusing on the photo-controlled performance of AZO with matching chain lengths in fatty acid systems.

Herein, we designed and synthesized a novel photo-controlled phase change composite material (C_14_Azo-MA), comprising myristic acid (MA) and a tailored azobenzene derivative, C_14_Azo, which possesses a chain length identical to that of MA. Compared with previously reported systems [8], this C_14_Azo-MA design offers distinct advantages. On the one hand, the extended alkyl chain length introduces stronger intermolecular van der Waals interactions. On the other hand, this strategy leads to superior thermal performance: the C_14_Azo-MA achieves significantly higher energy densities (up to 225.08 J g^−1^) and operates at elevated phase transition temperatures suitable for broader thermal management applications. The chemical structure of C_14_Azo-MA was thoroughly characterized using fourier transform infrared (FT-IR) spectroscopy, hydrogen nuclear magnetic resonance (^1^H NMR) spectroscopy, and X-ray diffraction (XRD). The photo-absorption and photoisomerization properties of C_14_Azo-MA were investigated by ultraviolet–visible (UV–Vis) spectroscopy. Thermal stability, optically-controlled phase change performance, and energy storage characteristics of C_14_Azo-MA were assessed using thermogravimetric analysis (TGA) and differential scanning calorimetry (DSC). The influence of C_14_Azo content on energy density and photo-induced supercooling effect were further investigated. Such C_14_Azo-MA materials open the way for the develop of advanced material for solar thermal storage applications.

## 2. Results and Discussion

### 2.1. Chemical Composition and Crystallinity

**Figure 1:**
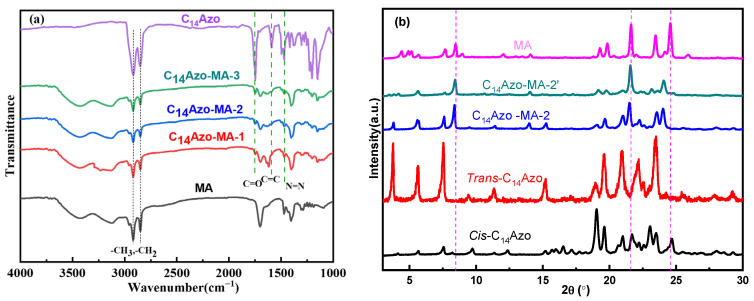
(**a**) FT-IR spectra and (**b**) XRD of C_14_Azo and C_14_Azo-MA.

In order to explore the chemical composition of C_14_Azo-MA, FTIR were conducted. Figure 1a displays the FTIR spectrum of C_14_Azo-MA, C_14_Azo and MA, where C_14_Azo-MA-1, C_14_Azo-MA-2 and C_14_Azo-MA-3 correspond to C_14_Azo mass fractions of 35, 45, and 55 wt% in C_14_Azo-MA composite, respectively. The FT-IR spectrum of C_14_Azo-MA includes characteristic peaks of both C_14_Azo and MA. Specifically, the characteristic peaks at 2918, 2850, and 1746 cm^−1^ indicate the presence of -CH_3_, -CH_2_, and -C=O stretching vibrations. The vibrational bands at 1590~1471 cm^−1^, and ~1416 cm cm^−1^ are attributed to the C=C of benzene and -N=N stretching vibrations, respectively. The peak at 1203 cm^−1^ corresponds to the asymmetric stretching vibration of the ester group. The vibrational band at 1249 cm^−1^ is ascribed to the bending vibration of -CH_2_. The characteristic peaks of the C–H bending (syn) and out-of-plane bending vibrations of -CH_2_- appear at ~1380 cm^−1^ and ~1226 cm^−1^, respectively [7]. The above results indicate the successful fabrication of the composite.

Additionally, the crystallinity of the C_14_Azo-MA (taking the C_14_Azo mass fractions of 45 wt% as an example) before and after irradiation were investigated by XRD and illustrated in Figure 1b. The non-UV-irradiated sample (C_14_Azo-MA-2) displayed diffraction peaks associated with MA chains [37,38] and *trans*-C_14_Azo [7]. In contrast, the UV-irradiated sample (C_14_Azo-MA-2′) revealed diffraction peaks corresponding to MA and *cis*-C_14_Azo, resulting from photoisomerization. Importantly, the maximum peak in C_14_Azo-MA-2′ is slightly shifted to a lower diffraction angle compared to the corresponding peak in the C_14_Azo-MA-2 sample, this is because the increased interlayer spacing of MA leads to weakened intermolecular interactions of MA. Additionally, the diffraction peak is broadened and its intensity reduced, resulting in the inhibition of MA crystallization and ultimately causing supercooling [8,34]. These results suggest that *cis*-C_14_Azo has a significant effect on the crystallinity of MA, which aids in extending its energy storage duration.

### 2.2. Photoisomerization Properties

**Figure 2:**
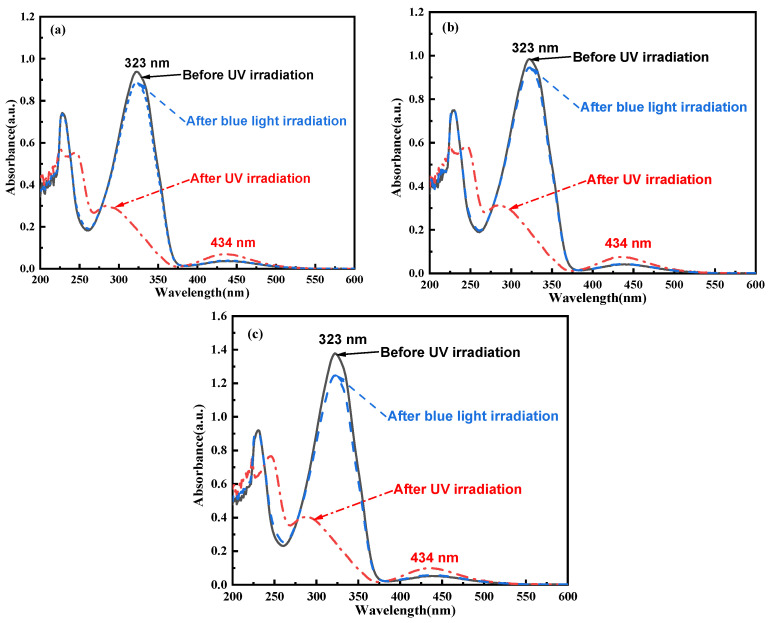
UV–Vis absorption spectra of (**a**) C_14_Azo-MA-1 (**b**) C_14_Azo-MA-2 and (**c**) C_14_Azo-MA-3 in DCM at room temperature before and after UV irradiation (365 nm, 20 mW cm^−2^) and blue irradiation (450 nm, 60 mW cm^−2^).

**Figure 3:**
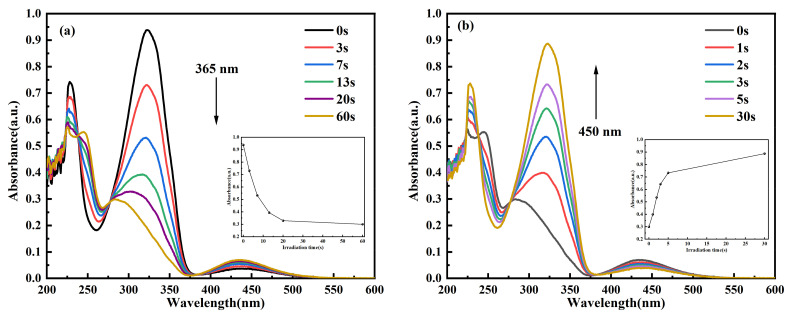

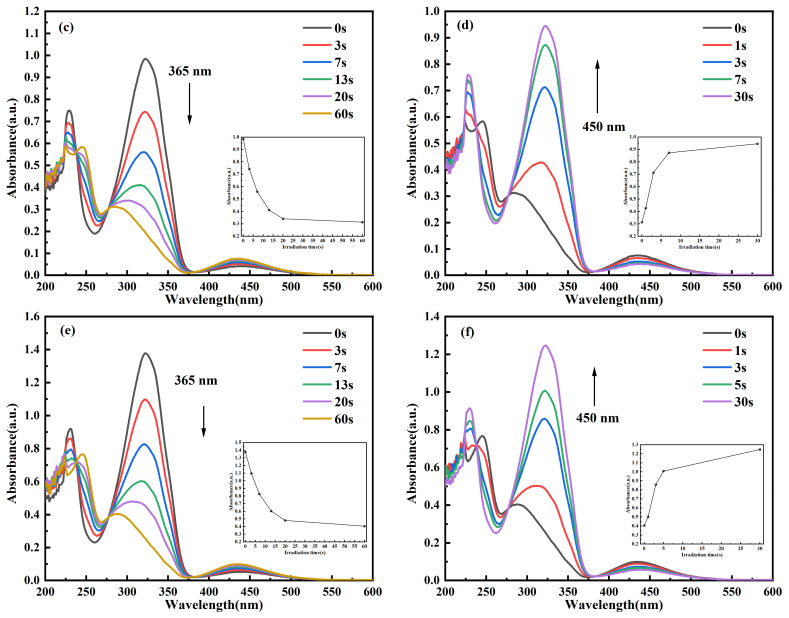
Time-dependent UV–Vis absorption spectra of C_14_Azo-MA in DCM at room temperature. (**a**) C_14_Azo-MA-1 (**c**) C_14_Azo-MA-2 and (**e**) C_14_Azo-MA-3 irradiated by 365 nm UV light with the evolution of Abs vs. time at 323 nm shown in the inset. (**b**) C_14_Azo-MA-1 (**d**) C_14_Azo-MA-2 (**f**) C_14_Azo-MA-3 irradiated by 450 nm blue light with the evolution of Abs vs. time at 323 nm shown in the inset.

The photoisomerization properties of C_14_Azo-MA are presented in Figure 2 and Figure 3. From the Figure 2, it can be seen that all *trans*-C_14_Azo-MA have a strong π→π* transition (323 nm) and a weak n→π* transition (434 nm) absorption bands in the range from 250 to 600 nm before UV irradiation. After UV irradiation (365 nm light), a decrease in the absorption intensity of the π→π* transition band was noted and a blue-shift from 323 nm to 283 nm was exhibited. In contrast, the absorption intensity of the n→π* transition band at 434 nm increased without any shift (dashed line). Following irradiation with blue light (450 nm), the absorption spectrum returns to its original spectral pattern (dashed dotted line). The spectral changes observed in C_14_Azo-MA indicate its excellent photoisomerization properties, reflecting the characteristics similar to the photochemical isomerization reactions of C_14_Azo [7,8], involving *trans*→*cis* and *cis*→*trans* isomerization transitions.

To further elucidate the time required to achieve the photostationary state (PSS) of *cis*↔*trans* C_14_Azo-MA, as well as the degree of isomerization, the photochemical isomerization behavior of C_14_Azo-MA was investigated by time-resolved UV–Vis absorption spectroscopy. As shown in Figure 3, this is the variation of absorbance of C_14_Azo-MA with irradiation time under UV and blue light. As the UV irradiation time increases, it can be observed that the intensity of the π-π* transition peak continuously decreases, while the intensity of the n-π* transition peak slightly increases until the PSS is reached (*trans*-rich→*cis*-rich), which takes 60 s. In other words, C_14_Azo-MA samples with varying C_14_Azo contents (35, 45, and 55 wt%) are able to reach the photostationary state after UV irradiation for 60 s. Subsequently, with the increase of blue light irradiation time, it can be found that the intensity of the π-π* transition peak continues to increase, while the intensity of the n-π* transition peak decreases slightly until it reaches PSS (*cis*-rich→*trans*-rich), and the time required to reach PSS is 30 s. These results indicate that C_14_Azo-MA has excellent photoisomerization performance. From a device perspective, such rapid conversion to PSS (60 s under UV and 30 s under blue light) implies potential for high-power-density applications, thereby achieving fast on-demand charging and fast heat release. However, practical designs must address the limitation of optical penetration depth in bulk materials, which favors the use of configurations such as thin films or porous structures to ensure uniform activation.

**Figure 4:**
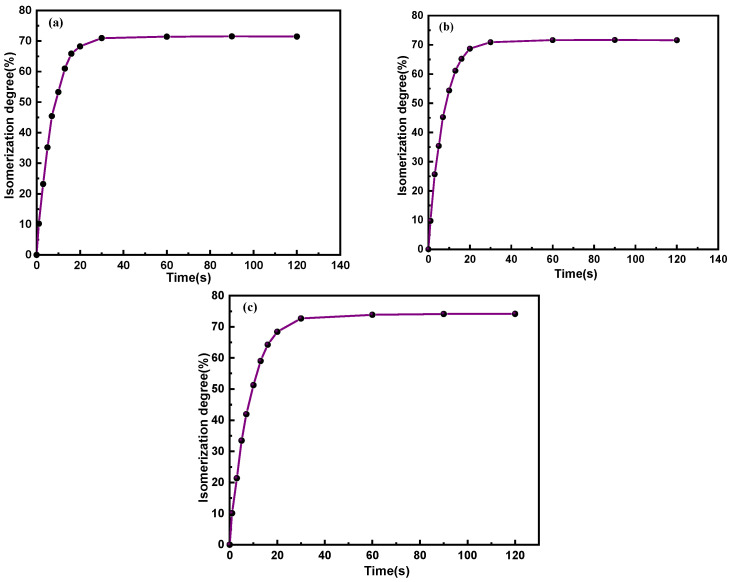
Isomerization degree of (**a**) C_14_Azo-MA-1, (**b**) C_14_Azo-MA-2 and (**c**) C_14_Azo-MA-3.

Additionally, the isomerization degree of C_14_Azo-MA, which is vital for energy storage, can be acquired by the formula presented in the literature [39] and plotted as shown in Figure 4. From the figure, it is evident that the amount of *cis*-isomer increases with increasing irradiation time under the same irradiation intensities, eventually reaching an isomerization degree of 71%, 71%, and 74% at the photostationary state, which are comparable with that of the corresponding C_14_Azo [7,8] and the current reports [40,41], showing that the C_14_Azo moiety maintains its favorable photoisomerization performance within the composite of C_14_Azo-MA.

Based on the aforementioned findings that C_14_Azo-MA possesses a high degree of isomerization and good photoreversibility, the kinetics of the photoinduced *trans*-to-*cis* and *cis*-to-*trans* isomerization processes for C_14_Azo-MA in DCM solution were quantitatively studied under 365 nm and 450 nm light irradiation. The first-order rate constants for isomerization were calculated using Equation (1) [42].

ln[(*A*_∞_ − *A*_t_)/(*A*_∞_ − *A*_0_)] = −*kt* (1)

where *A*_0_, *A*_t_, and *A*_∞_ refer to the absorbance values of the π-π* transition at 323 nm at initial moment (time zero), a given time *t*, and infinite time, respectively. The variable *k* signifies the first-order rate constant for the photoinduced isomerization process.

**Figure 5:**
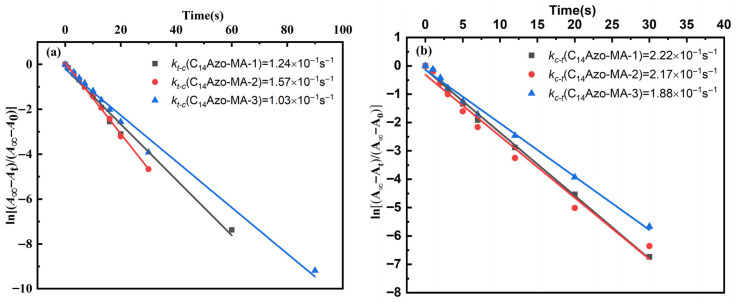
First-order plots for (**a**) *trans*-*cis* and (**b**) *cis*-*trans* isomerization processes of C_14_Azo-MA.

The first-order kinetic plots detailing photoinduced *trans*-to-*cis* and *cis*-to-*trans* isomerization of C_14_Azo-MA in DCM solution are shown in Figure 5. It can be observed that C_14_Azo-MA with varying C_14_Azo contents displays similar linear behavior, conforming to first-order kinetics. And the first-order rate constant for C_14_Azo-MA with varying C_14_Azo contents are all on the same order of magnitude, exhibiting the outstanding photoisomerization performance.

### 2.3. Thermal Stability and Cyclic Stability

**Figure 6:**
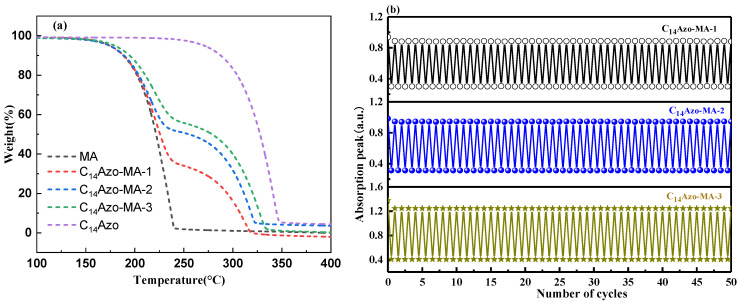
(**a**) TGA curves and (**b**) cyclic stability of C_14_Azo-MA.

Thermal stability is one of the important performance indicators for evaluating the effectiveness and durability of materials in practical energy storage applications. Figure 6a presents the TGA curves for C_14_Azo, pure MA, and C_14_Azo-MA with different C_14_Azo contents. The curves reveal that all C_14_Azo-MA samples exhibit a two-step weight loss process, while pure C_14_Azo and MA exhibit only a single-step degradation. For C_14_Azo-MA, the former step is attributed to the bond cleavage of carbon chains in the MA component [43,44], while the latter is due to the breakage of the bonds between the nitrogen atoms of the -N=N- group and the carbon atoms of the phenyl rings in C_14_Azo [7,8]. Furthermore, the thermal stability of C_14_Azo-MA is slightly enhanced with increasing C_14_Azo content in the temperature range of 250 to 400 °C. This is because the degradation temperature of C_14_Azo is higher than that of MA, and the relative content of MA decreases as the C_14_Azo content increases. Overall, the C_14_Azo-MA showed good thermal stability up to 200 °C.

In addition, the cycling stability of C_14_Azo-MA was investigated through repeated 365 nm light-irradiated and 450 nm light-irradiated cycles based on the *trans*-*cis*-*trans* isomerization process. The *trans*-C_14_Azo-MA was converted to *cis*-C_14_Azo-MA upon UV light irradiation, and subsequently reverts back to the *trans*-form upon exposure to blue light. Figure 6b displays the absorbance changes (323 nm) during the *trans*-form→*cis*-form→*trans*-form transitions, as recorded by UV–Vis spectroscopy. The results demonstrate that C_14_Azo-MA possesses excellent cycling stability over 50 cycles, with no significant fatigue or degradation.

### 2.4. Optically-Controlled Phase Change Performance

**Figure 7:**
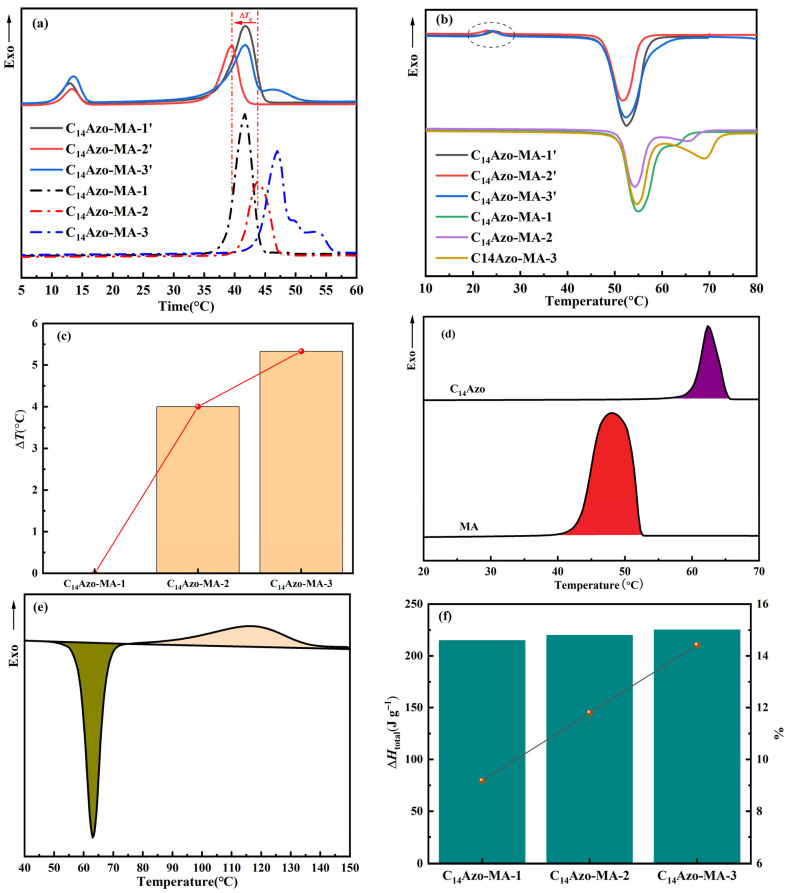
Energy storage performance of C_14_Azo-MA. (**a**) DSC solidification plots of irradiated and unirradiated C_14_Azo-MA by UV. (**b**) DSC melting plots of irradiated and unirradiated C_14_Azo-MA by UV. (**c**) Δ*T*_c_ of C_14_Azo-MA. (**d**) DSC exothermic heat of C_14_Azo and MA in the cooling stage. The purple part and red one show the crystallization of C_14_Azo and MA respectively. (**e**) DSC exothermic heat of C_14_Azo during *cis*→*trans* isomerization. The olive green part shows the melting of *cis*-C_14_Azo, while the light peach part shows the isomerization change of *cis*-C_14_Azo→*trans*-C_14_Azo. (**f**) Summary of ∆*H*_total_ for C_14_Azo-MA in the bar chart and their enhancements over MA in the point and line plot.

The crystallization temperature (*T*_c_) is widely recognized as a key parameter in practical energy storage application. The *T*_c_ of the *trans*- and *cis*-C_14_Azo-MA were measured by DSC, as shown in Figure 7a. Almost all of *trans*-C_14_Azo-MA samples exhibit a single peak, which is attributed to the formation of a eutectic between the *trans*-C_14_Azo and MA [8,31]. Notably, when the content of *trans*-C_14_Azo is 55 wt%, the crystallization range of C_14_Azo-MA is broader than that of other compositions. Besides, it can be observed that as the C_14_Azo content increases, the crystallization peak of C_14_Azo-MA shifts to higher temperatures. However, two crystallization peaks are observed for almost all *cis*-C_14_Azo-MA, corresponding to the individual crystallization of *cis*-C_14_Azo and MA (*T*_c_ ≈ 48 °C), which is clearly different from the case of *trans*-C_14_Azo-MA. This result, which is in agreement with the aforementioned XRD data, can be attributed to the enhanced interaction between *cis*-C_14_Azo and MA, specifically the formation of hydrogen bonds-a phenomenon absent in the *trans*-C_14_Azo-MA system (circled in Figure 7b), leading to a concomitant weakening of the inherent interactions of MA. Consequently, such an effect results in the *T*_c_ of MA to differ between the two systems at the same C_14_Azo content.

In addition, Δ*T*_c_ between *trans*-C_14_Azo-MA and *cis*-C_14_Azo-MA under different C_14_Azo content was comprehensively analyzed to further elucidate the optically-controlled phase change performance of C_14_Azo-MA. As shown in Figure 7c and Table 1, the Δ*T*_c_ of C_14_Azo-MA increases as the C_14_Azo content increases. This phenomenon can be attributed to the accumulation of larger *cis*-C_14_Azo in the restricted MA. With the increase of C_14_Azo content, the steric hindrance imposed by bent *cis*-C_14_Azo becomes more significant, which significantly destroys the ordered arrangement of MA molecules and inhibits the nucleation process, resulting in an increase in Δ*T*_c_ value. A maximum Δ*T*_c_ of 5.33 °C is observed between C_14_Azo-MA-3 and C_14_Azo-MA-3′. The study indicates that this temperature difference can be effectively adjusted through the optimization of C_14_Azo content. More importantly, this adjustable Δ*T*_c_ has important practical significance in practical thermal management. A large supercooling effect (Δ*T*_c_ ≈ 5.33 °C) allows the latent heat to be locked in a metastable liquid state and released when needed, thereby avoiding spontaneous heat loss. In addition, the phase transition characteristics of these composites are highly compatible with the needs of low-grade energy harvesting and solar thermal storage. In these applications, controlled heat release is essential to maximize energy efficiency.

### 2.5. Energy Storage Performance and Durability Assessment

Energy density stands as a crucial metric for energy storage capabilities of OPCMs. The overall energy density of C_14_Azo-MA comprises both the latent heat of phase change and the enthalpy of isomerization, and can be determined by Equation (2) [8,34].

∆*H*_total_ = χ∆*H*_MA_ + (1 − χ)∆*H_trans_*_-C14Azo_ + (1 − χ)∆*H*_iso(C14Azo)_ (2)

where ∆*H*_total_ represents the total heat released during the *cis*→*trans* isomerization and phase transition of C_14_Azo-MA. ∆*H*_MA_ and ∆*H_trans_*_-C14Azo_ corresponds to is the crystallization enthalpy of MA and *trans*-C_14_Azo, respectively. ∆*H*_iso(C14Azo)_ denotes the isomerization enthalpy of C_14_Azo, and χ is the content (mass fraction) of MA in C_14_Azo-MA.

DSC analysis was conducted to determine ∆*H*_MA_, ∆*H_trans_*_-C14Azo_, and ∆*H*_iso(C14Azo)_ for the calculation of ∆*H*_total_. From Figure 7d,e, ∆*H*_MA_, ∆*H_trans_*_-C14Azo_, and ∆*H*_iso(C14Azo)_ were found to be 196.71 J g^−1^, 142.70 J g^−1^, and 105.59 J g^−1^, respectively. Using Equation (2), these values were calculated and plotted as the bar chart in Figure 7f, which shows that the ∆*H*_total_ of C_14_Azo-MA rises as C_14_Azo content increases. Ultimately, the ∆*H*_total_ obtained for C_14_Azo-MA-1, C_14_Azo-MA-2, and C_14_Azo-MA-3 were 214.76 J g^−1^, 219.92 J g^−1^, and 225.08 J g^−1^, respectively, corresponding to enhancements of 9.18%, 11.80%, and 14.42% over MA (as shown in the point and line plot in Figure 7f). This enhancement in ∆*H*_total_ is mainly due to the strengthened intermolecular interactions as well as the extra photochemical energy stored by the C_14_Azo compound. The result indicates that the C_14_Azo-MA shows higher density energy than other AZO-fatty acid systems previously reported [31,34,35] and is comparable to AZO-OPCMs for solar energy storage application [7,8]. To explicitly highlight these advantages, a comprehensive comparison of key thermal metrics, including total enthalpy (∆*H*_total_), crystallization temperature (*T*_c_), and photoinduced crystallization temperature difference (Δ*T*_c_), is presented in Table 2. As summarized in the table, the C_14_Azo-MA achieves a superior energy density of 225.08 J g^−1^ through the chain-length matching strategy, surpassing the typical values observed in mismatched counterparts. This incremental yet significant gain confirms the effectiveness of optimizing molecular interactions for high-performance thermal storage.

**Figure 8:**
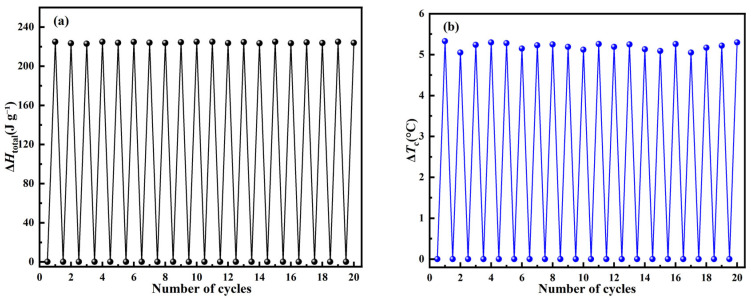
Durability assessment of C_14_Azo-MA (**a**) ∆*H*_total_ and (**b**) Δ*T*_c_.

In addition to the aforementioned UV–Vis level cycle stability, DSC level cycle stability is also a performance index to determine the durability of C_14_Azo-MA. The durability of C_14_Azo-MA was investigated through repeated DSC measurements involving 365 nm light irradiation and heating/cooling. C_14_Azo-MA-3 was selected as samples for durability testing due to its superior performance in optically-controlled phase transitions. The C_14_Azo-MA-3 in the *trans*-rich state was subjected to 365 nm UV light, transforming it into the *cis*-rich state. The subsequent reversion from *cis*-rich to *trans*-rich state was achieved through heating using DSC. Figure 8a,b shows the changes in Δ*H*_total_ and Δ*T*_c_ from *trans*-rich to *cis*-rich to *trans*-rich state, respectively. The Δ*H*_total_ of sample C_14_Azo-MA-3 is 223.56 J g^−1^–225.08 J g^−1^ and the Δ*T*_c_ of sample C_14_Azo-MA-3 is 5.05 °C–5.33 °C. The result shows an excellent durability of C_14_Azo-MA over 20 cycles without noticeable attenuation.

## 3. Materials and Methods

### 3.1. Preparation of C_14_Azo-MA

All the main materials used without further purification were obtained from commercial sources, unless otherwise mentioned. The C_14_Azo molecule was synthesized according to our previous work [8] (^1^H NMR, 400 MHz, CDCl_3_-d): δ (ppm) = 7.96 (m, 2H), 7.91 (m, 2H), 7.50 (m, 3H), 7.25 (m, 2H), 2.59 (t, *J* = 7.5 Hz, 2H), 1.78 (m, 2H), 1.32 (m, 16H), 0.89 (t, *J* = 6.8 Hz, 3H).

C_14_Azo-MA was prepared by the melting method. Specifically, C_14_Azo was mixed with myristic acid (MA) at mass fractions of 35 wt%, 45 wt%, and 55 wt%, respectively. The mixture was heated until completely melted, followed by mechanical stirring for 30 min. After natural cooling, the C_14_Azo-MA composite materials (*trans*-C_14_Azo-MA) were obtained and labeled as C_14_Azo-MA-1, C_14_Azo-MA-2, and C_14_Azo-MA-3, respectively. The solid-state C_14_Azo-MA composites were then transferred onto glass substrates for further ultraviolet (UV) irradiation. After irradiation, the materials (*cis*-C_14_Azo-MA) were labeled as C_14_Azo-MA-1′, C_14_Azo-MA-2′, and C_14_Azo-MA-3′, respectively.

### 3.2. Characterizations

The ^1^H NMR (400-MHz, AVANCE IIITM HD, Bruker, Mannheim, Germany) of C_14_Azo were tested using a nuclear magnetic resonance spectrometer, with deuterated chloroform (CDCl_3_-d) as the solvent and testing ranges of 0–20 ppm. A FT-IR (Nicolet 6700, Thermo Fisher Scientific, Waltham, MA, USA) was used to characterize the vibration modes of the C_14_Azo and C_14_Azo-MA group, with the sample prepared using potassium bromide pellets and a testing range of 4000–400 cm^−1^. XRD (Ultima IV, Rigaku, Tokyo, Japan) patterns were obtained by Cu Kα radiation (*k* = 0.15 nm) at a scanning rate of 5.0 min^−1^.

The photoisomerization properties of C_14_Azo-MA in DCM solution using 1 cm pathlength quartz cuvettes were characterized using UV–Vis (UV-1900i, Shimadzu, Kyoto, Japan) with UV (365 nm) light intensity of 20 mW cm^−2^ and blue (450 nm) light intensity of 60 mW cm^−2^. Subsequently, the samples were irradiated with a light source of a specified wavelength until a photostationary state (PSS) was reached, that is, no change in absorbance was observed.

The thermal stability of C_14_Azo-MA was tested using TGA (SDT Q600, TA Instruments, Newcastle, DE, USA) in a nitrogen atmosphere, within a temperature range of 25–400 °C, at a heating rate of 10 °C min^−1^ and a gas flow rate of 50 mL min^−1^. The *T*_c_ and energy density of C_14_Azo-MA were measured using DSC (DSC3, Mettler Toledo, Zurich, Switzerland) under a nitrogen atmosphere, with the heating/cooling rate set at 20 °C min^−1^. The process is shown as follows. Solid C_14_Azo-MA with different C_14_Azo content were heated at 59 °C to absorb external thermal energy, and then irradiated by a UV lamp (365 nm, 80 mW cm^−2^) to store optical energy. After 1 h of irradiating, the samples were transferred to DSC pans in the dark for DSC test.

## 4. Conclusions

A composite material, C_14_Azo-MA, was successfully prepared by doping MA with C_14_Azo featuring an identical carbon chain length, designed for solar energy storage and controlled release. The composite was found to preserve the robust photoisomerization properties of the C_14_Azo dopant. The incorporation of these photo-responsive C_14_Azo molecules induces tunable supercooling and modifies the crystallization behavior of the system. Additionally, the supercooling degree and energy density can be tuned by adjusting the mass fractions of C_14_Azo in the composite, allowing for stable thermal energy storage below the intrinsic freezing point of the MA. Specifically, the Δ*T*_c_ and ∆*H*_total_ of C_14_Azo-MA increases as the C_14_Azo content increases. Eventually, the maximum supercooling degree reached is 5.33 °C. By synergistically storing both latent heat of crystallization and isomerization energy, the C_14_Azo-MA composite achieves a remarkable energy density of 225.08 J g^−1^, which surpasses that of pure MA by 14.42%. The system also maintained excellent stability. This study opens a new avenue for designing high-capacity photothermal storage systems for solar energy storage applications.

## Figures and Tables

**Table 1:** The *T*_c_ and Δ*T*_c_ of C_14_Azo-MA with different C_14_Azo content.

Code	Crystalization		
	*T*_c_ (°C)		Δ*T*_c_ (°C)
	Before UV Irradiation (*Trans*-Rich)	After UV Irradiation (*Cis*-Rich)	
C_14_Azo-MA-1/C_14_Azo-MA-1′	41.67	41.67	-
C_14_Azo-MA-2/C_14_Azo-MA-2′	43.67	39.67	-
C_14_Azo-MA-3/C_14_Azo-MA-3′	47	41.67	-
C_14_Azo-MA-1-C_14_Azo-MA-1′ ^a^	-	-	0
C_14_Azo-MA-2-C_14_Azo-MA-2′ ^b^	-	-	4
C_14_Azo-MA-3-C_14_Azo-MA-3′ ^c^	-	-	5.33

^a–c^ present the Δ*T*_c_ of the corresponding C_14_Azo-MA before and after UV irradiation.

**Table 2:** A comparison of ∆*H*_total_, *T*_c_ and Δ*T*_c_ of azobenzene-OPCM systems from the latest reports.

Azobenzene-OPCM	∆*H*_total_ (J g^−1^)	*T*_c_ (°C)	Δ*T*_c_ (°C)
PCC [7]	239	25.66	7.67
AZO-A-14/CA12 [31]	193.73	/	9.19
Optically-controlled PCM [34,35]	200	28	10
AZO-OPCC [8]	217.72	25	12
C_14_Azo-MA (this study)	225.08	41.67	5.33

## Data Availability

The data supporting the findings of this study are available within the article.

## Abbreviations

The following abbreviations are used in this manuscript:

OPCMs	Organic phase change materials
AZO	Azobenzene
Δ*H*_iso_	Isomerization enthalpy
Δ*T*_c_	Photoinduced crystallization temperature difference
MA	Myristic acid
C_14_Azo	Azobenzene derivative
C_14_Azo-MA	Azobenzene-based organic phase change composite
FT-IR	Fourier transform infrared
^1^HNMR	Proton nuclear magnetic resonance
XRD	X-ray diffraction
UV–Vis	UV–visible
TGA	Thermogravimetric analyzer
DSC	Differential scanning calorimeter
DCM	Dichloromethane
*A*_0_	The absorbance values of the π-π* transition at 323 nm at initial moment (time zero)
*A*_t_	The absorbance values of the π-π* transition at 323 nm at a given time *t*
*A*_∞_	The absorbance values of the π-π* transition at 323 nm at infinite time
*k*	First-order kinetic constants
*k*_t-c_	First-order rate constants from *trans* to *cis*
*k*_c-t_	First-order rate constants from *cis* to *trans*
*T*_c_	Crystallization temperature
∆*H*_total_	The total heat released during the *cis*→*trans* isomerization and phase transition of C_14_Azo-MA
∆*H*_MA_	The crystallization enthalpy of MA
∆*H_trans_*_-C14Azo_	The crystallization enthalpy of *trans*-C_14_Azo
∆*H*_iso(C14Azo)_	The isomerization enthalpy of C_14_Azo
χ	The content (mass fraction) of MA in C_14_Azo-MA
